# Discussion of Methods in Berlin for Fighting the Hoof- and Mouth-disease

**Published:** 1898-05

**Authors:** 


Discussion of Methods in Berlin for Fighting the Hoof- and Mouth-disease.—On January 14th a conference of landed proprietors, veterinarians, and bacteriologists was held in the imperial health-office to discuss methods of investigation and means of defence against hoof- and mouth-disease, which has so much injured the German stock-raiser. The director, privy-councillor Dr. Kohler, presided. As a basis of discussion, the results of the experimental investigations of two commissions were presented: those made in the health department itself in regard to the disease, and those made since April of last year in the Prussian Institute for Infective Diseases. Among those present were the Minister of State, Count Zedlitz, as a landed proprietor; as veterinarians, Profs. Schutz and Eggeling from Berlin, Drs. Lydtin (Baden-Baden), Gornig (Munich), and Vollers (Hamburg); as bacteriologists, Dr. Löffler (Greifswald) and chief staff-physician Wersser (Berlin).—*Idem.*

